# Dynamical universality and vibrational divergence in 2D supercooled liquids, quasicrystals, and crystals

**DOI:** 10.1039/d5sm00870k

**Published:** 2026-05-28

**Authors:** Edwin A. Bedolla-Montiel, Marjolein Dijkstra

**Affiliations:** a Soft Condensed Matter & Biophysics, Debye Institute for Nanomaterials Science, Utrecht University, Princetonplein 1 3584 CC Utrecht Netherlands m.dijkstra@uu.nl e.a.bedollamontiel@uu.nl

## Abstract

We investigate the dynamical behavior and vibrational properties of three structurally distinct two-dimensional systems: a supercooled binary liquid, a dodecagonal quasicrystal (DDQC), and a hexagonal crystal. Using molecular dynamics simulations, we find that all three systems exhibit transient caging in the mean-squared displacement and non-Gaussian single-particle displacement statistics. However, the temperature dependence of the dynamics differs markedly among them. In the supercooled liquid, the peak of the non-Gaussian parameter increases upon cooling, reflecting the growth of dynamical heterogeneity. In contrast, in the DDQC, the peak decreases as temperature is lowered, consistent with the progressive suppression of thermally activated, localized rearrangements. For the DDQC, this behavior is confirmed by the cage-relative self part of the van Hove function, which shows a systematic suppression of large single-particle displacements upon cooling. At the same time, the DDQC exhibits a large dynamical susceptibility, indicating that many-body dynamical correlations remain strong despite the reduction of large particle displacements upon cooling. A real-space cluster analysis reveals that mobile particles remain organized into extended, spatially correlated, dynamical clusters, with temperature primarily affecting the cluster-size distribution rather than the intrinsic cluster morphology. The vibrational spectra further differentiate the three systems: the crystal exhibits van Hove singularities, the supercooled liquid shows a boson peak, and the DDQC displays additional low-frequency contributions associated with quasiperiodic order. These results establish the DDQC as an intermediate state, combining glass-like caging dynamics with vibrational signatures strongly influenced by quasiperiodic order.

## Abstract 2

Dodecagonal quasicrystals show cage-trapping dynamics like supercooled liquids, yet their vibrational modes display phase coherence and defect signatures akin to periodic crystals, a hybrid state bridging amorphous and ordered solids.

## Graphical abstract

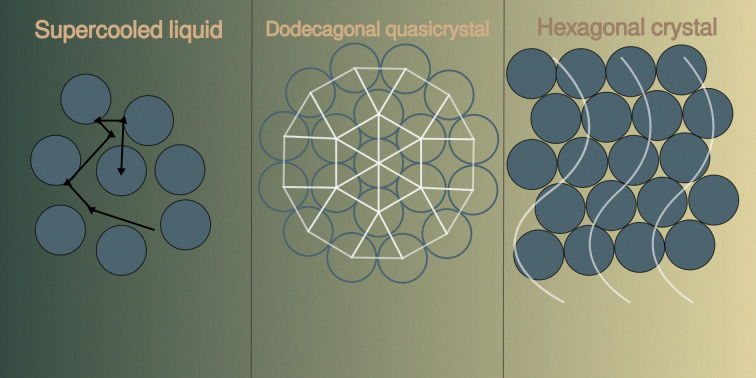

## 1 Introduction

At the intersection of ordered and disordered matter lie two captivating states of matter: quasicrystals (QCs), with their nonperiodic but long-range order, and supercooled liquids approaching the glass transition. Despite their fundamentally different structural organizations, these systems exhibit striking dynamical similarities that challenge our traditional understanding on the relationship between structure and dynamics. A related perspective was offered by Angell,^1^ who demonstrated that amorphous materials can undergo transitions analogous to (first-order) crystallization, resulting in distinct glassy states with unique thermodynamic signatures. He related this behavior to QCs, whose nonperiodic, unconventional ordering blurs the boundary between disordered and crystalline phases.

Quasicrystals exhibit long-range orientational order without translational periodicity. First discovered in metallic alloys by Shechtman *et al.*,^2^ QCs have since attracted growing interest in soft matter, particularly following their experimental and computational realization in colloidal, polymeric, and molecular systems.^3–15^ These structures have attracted particular interest for photonic applications, where their aperiodic order enables unique transmission characteristics, band gaps, and disorder-enhanced transport phenomena.^16–19^

Supercooled liquids and glasses have long been central to the study of complex condensed matter dynamics. These systems exhibit hallmark phenomena such as dynamical heterogeneity,^20,21^ transient caging,^22–24^ and cooperative particle rearrangements.^25–27^ As temperature decreases, supercooled liquids exhibit a pronounced dynamical slowdown that eventually leads to the glass transition, where molecular motion becomes severely constrained in the absence of crystallization.^28^ Unlike crystals or quasicrystals, their structural complexity emerges not from long-range order but from disordered local rearrangements and medium-range correlations that strongly influence the dynamics.

Despite extensive research on QCs and supercooled liquids as separate systems, comparative studies that explore their structural and dynamical similarities remain limited. Recent work by Cao *et al.*^29^ demonstrated that three-dimensional icosahedral QCs and supercooled liquids exhibit comparable phonon dynamics. However, a systematic comparison of structure-dynamics relationships, vibrational properties, and topological features in two-dimensional (2D) systems has yet to be undertaken. The apparent paradox that structurally distinct systems can display remarkably similar dynamical behavior motivates the present study. We focus on 2D systems, which offer distinct advantages: enhanced accessibility for visualizing structural motifs and collective motions, and direct relevance to experimental colloidal systems where such behaviors can be observed in real time and real space.^30^

Previous studies have extensively examined structure-dynamics relationships in both glasses and quasicrystals, though largely in isolation. In glasses, dynamical heterogeneity has been closely linked to local structural ordering, with particular emphasis on identifying structural motifs that correlate with particle mobility.^31,32^ Medium-range structural features have also been associated with cooperative rearrangements and string-like motion.^25,26^ In QCs, research has primarily focused on phason dynamics,^33,34^ particle flips,^35^ and self-diffusion mechanisms.^36,37^ Notably, Zhao *et al.*^37^ proposed that QCs may represent an intermediate state between crystals and glasses in terms of their dynamic properties, although their focus was on diffusion rather than vibrational or topological characteristics. Beyond a recent comparative study in three-dimensional systems,^29^ systematic investigations of dynamical heterogeneity, vibrational properties, and associated topological features in two-dimensional QCs and glasses have received limited attention.

In this work, we employ molecular dynamics simulations to systematically compare the structural and dynamical properties of a binary supercooled liquid, a two-dimensional dodecagonal quasicrystal (DDQC), and a hexagonal crystal. To model these phases, we utilize two distinct interaction potentials: both the DDQC and the hexagonal crystal are stabilized using the same continuous square-shoulder-like potential, whereas the supercooled liquid is modeled as a binary mixture interacting *via* a soft repulsive pair potential. To mitigate the challenges posed by Mermin–Wagner fluctuations inherent to two-dimensional systems, we implement cage-relative analysis techniques for all dynamical observables.^38^ We quantify dynamical heterogeneity using the non-Gaussian parameter and examine the spatial extent of collective motion *via* the dynamical susceptibility. To elucidate the microscopic origin of the DDQC dynamics, we complement these observables with cage-relative single-particle displacement distributions and a real-space analysis of mobile-particle clustering. We further investigate the vibrational properties by computing the dynamical matrix of energy-minimized configurations and extracting the vibrational density of states and participation ratios.

Our analysis reveals that the dodecagonal quasicrystal exists as a unique hybrid state, bridging the gap between ordered solids and disordered glass-formers. While all three systems exhibit cage-trapping plateaus and pronounced non-Gaussian behavior, their temperature-dependence dynamics differ markedly. In the supercooled liquid, the non-Gaussian parameter increases upon cooling, reflecting increasing heterogeneous single-particle dynamics. In the hexagonal crystal, the relatively large non-Gaussian parameter is consistent with sparse, defect-mediated intermittent hopping events. These events produce broad tails in the single-particle displacement distribution but do not necessarily generate large, spatially correlated dynamical regions. In other words, the crystal can exhibit pronounced single-particle intermittency without developing the extended collective mobile regions that would yield a correspondingly large dynamic susceptibility. More intriguingly, the DDQC shows unusual dynamical behavior. Upon entering the slow-dynamics regime, the non-Gaussian parameter decreases while the dynamical susceptibility increases upon cooling. We thus observe a decoupling between single-particle intermittency and many-body correlations: upon cooling, large local particle displacements become less frequent, leading to a decrease in the non-Gaussian parameter, while the mobility field remains spatially correlated over extended regions, keeping the dynamical susceptibility large.

The paper is organized as follows. In Section 2, we describe the model systems and simulation details. We analyze the resulting dynamical behavior and heterogeneity in Section 3. Section 4 presents a DDQC-specific real-space analysis of this dynamical decoupling. Section 5 examines the vibrational properties through the density of states and participation ratios. Finally, Section 6 summarizes our findings and provides an outlook.

## 2 Model systems and simulation details

We performed molecular dynamics (MD) simulations on a supercooled binary liquid mixture, a two-dimensional dodecagonal quasicrystal (DDQC), and a hexagonal crystal using the LAMMPS software package.^41,42^ Equilibration runs were carried out in the canonical (NVT) ensemble for systems of *N* = 4096 particles. Production runs for measuring dynamical and vibrational properties were performed in the microcanonical (NVE) ensemble. Periodic boundary conditions were applied in all spatial directions.

### 2.1 2D supercooled binary liquid mixture

For the supercooled liquid, we employ an equimolar binary mixture of particles with diameters *σ*_S_ and *σ*_L_, interacting *via* a Weeks–Chandler–Andersen potential. This interaction potential between species *α*,*β* = L,S is defined as1
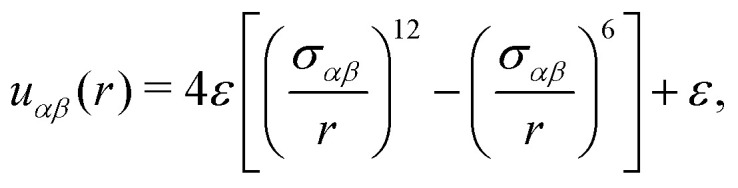
where *σ*_*α*,*β*_ = (*σ*_*α*_ + *σ*_*β*_)/2 represents the (cross-)interaction term, making the mixture additive. The constant *ε* ensures that the interaction potential smoothly vanishes at the cutoff radius, *u*_*α*,*β*_(*r*_cut_) = 0, with *r*_cut_ = 2^1/6^*σ*_*α*,*β*_, making the potential purely repulsive and continuous. The continuous nature of this potential makes it particularly well suited for analyzing vibrational properties, in contrast to discontinuous potentials such as the hard-sphere potential, which require specialized techniques for such analyses.^43^ The size ratio is set to 
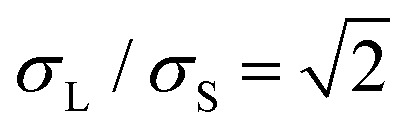
 to introduce size disparity and suppress crystallization.^44–47^ The particle masses follow *m*_L_/*m*_S_ = (*σ*_L_/*σ*_S_)^2^. The control parameter is the reduced temperature *k*_B_*T*/*ε*, where *T* is the absolute temperature and *k*_B_ denotes Boltzmann's constant. All simulations are performed at a fixed number density of *ρσ*^2^ = 0.75.

We performed MD simulations on the supercooled binary liquid mixture using an integration time step of Δ*t* = 0.001*τ*_G_, where 
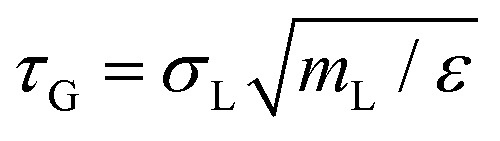
 represents the MD time unit for the glass. We employ the Bussi–Donadio–Parrinello thermostat to keep the temperature fixed^48^ using a damping parameter of 0.1*τ*_G_.

The system was first equilibrated at a high temperature of *k*_B_*T*/*ε* = 5 for 10^3^*τ*_G_ (equivalent to 10^6^ MD time steps), then linearly cooled over 10^4^*τ*_G_ (10^7^ MD time steps) to a target temperature *k*_B_*T*/*ε* ∈ [1.12,1.24], which corresponds to a temperature range above the mode-coupling-theory (MCT) temperature as reported in ref. 47. At the target temperature, the system was equilibrated for 2 × 10^6^*τ*_G_ (2 × 10^9^ MD time steps) to ensure thermal and structural stability. This corresponds to approximately 75 to 10^3^ structural relaxation times *τ*_*α*_ across the studied temperature range, where *τ*_*α*_ is defined by *q*(*τ*_*α*_) = 1/*e* (see Fig. 4a). We use five independent samples to obtain all reported quantities.

**Fig. 1:**
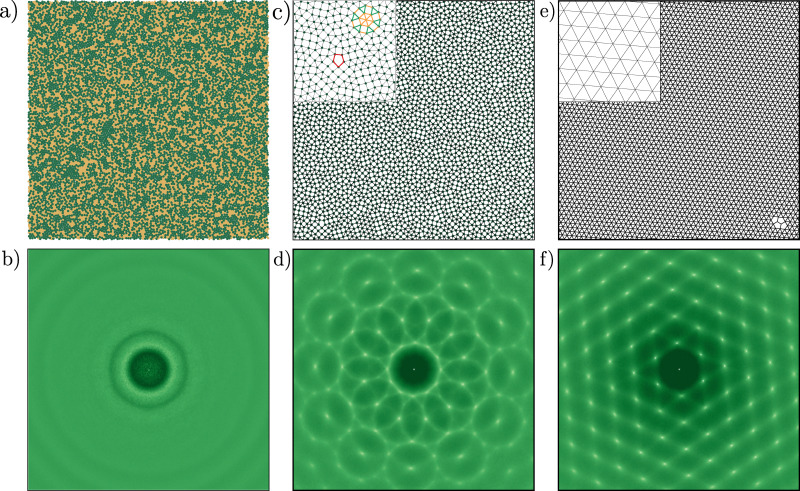
Typical configurations of all three studied systems in this work: (a) a supercooled binary liquid mixture; (c) a dodecagonal quasicrystal; and (e) a hexagonal crystal. All systems are composed of *N* = 4096 particles. Interparticle bonds (black lines) are drawn for particle distances *r* < 1.4*σ* for the dodecagonal quasicrystal and the hexagonal crystal. The inset in panel (c) highlights a characteristic dodecagonal “wheel” motif in orange and green—an essential motif of a true quasicrystal formed with a random square-triangle tiling.^39,40^ The inset also shows in red a defect structure, frequently observed in soft-matter quasicrystals. In the lower row, intensity maps of the static structure factor *S*(*k*) are shown for: (b) supercooled liquid; (d) dodecagonal quasicrystal; (f) hexagonal crystal.

Fig. 1a shows a typical configuration of the supercooled liquid, along with the corresponding intensity map of the static structure factor in Fig. 1b. The structure factor displays diffuse concentric rings, consistent with the absence of long-range structural order characteristic of amorphous systems.

### 2.2 2D dodecagonal quasicrystal

To model a two-dimensional dodecagonal quasicrystal (DDQC), we employ a continuous interaction potential designed to mimic the behavior of a square-shoulder potential. The potential is given by2
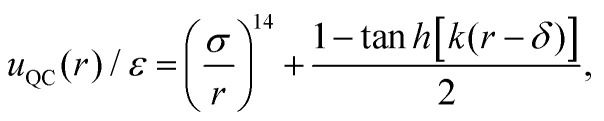
where *ε* sets the energy scale, *σ* represents the typical core diameter, *kσ* = 10 controls the steepness of the repulsive shoulder, and *δ* = 1.35*σ* determines the interaction range. The parameters *k* and *δ* are carefully tuned to stabilize a DDQC phase;^49,50^ varying these values can yield QCs with different orientational symmetries. All simulations were performed at a fixed number density of *ρσ*^2^ = 0.94, selected based on previously reported phase diagrams,^49,51^ and the control parameter is the dimensionless temperature *k*_B_*T*/*ε*. We employed the Bussi–Donadio–Parrinello thermostat^48^ with a damping parameter of 0.1 *τ*_QC_ to control the temperature. The stability region for the DDQC is very narrow, a feature also demonstrated for a binary DDQC mixture.^52^ We performed MD simulations on the DDQC using an integration time step of Δ*t* = 0.005 *τ*_QC_, where 
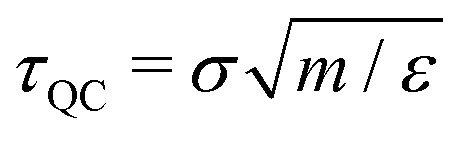
 denotes the MD time unit with *m* the particle mass.

The DDQC was generated using an annealing protocol similar to that of the supercooled liquid.

Initially, the system was equilibrated at a high temperature of *k*_B_*T*/*ε* = 1 for at least 5 × 10^3^*τ*_QC_ (10^6^ MD time steps). Subsequently, the temperature was reduced using a linear cooling rate over 5 × 10^4^*τ*_QC_ (10^7^ MD time steps) to reach a target temperature range *k*_B_*T*/*ε* ∈ [0.16,0.185], within which the DDQC is thermodynamically stable.^49,51^ Once the target temperature was reached, the DDQC was further equilibrated for 2.5 × 10^6^*τ*_QC_ (5 × 10^8^ MD time steps) to ensure the formation and stability of the quasicrystalline structure, corresponding to approximately 6–60 structural relaxation times *τ*_*α*_ across the temperature range studied (see Fig. 4c). We use five independent samples to obtain all reported quantities.

An exemplary configuration of the DDQC is shown in Fig. 1c, where the structure exhibits the characteristic random square-triangle tiling that constitutes the typical motifs of the quasicrystalline structure. The inset highlights the dodecagonal wheel, a hallmark structural unit of square-triangle quasicrystals, commonly observed in all true QCs formed from the square-triangle random tiling.^39,40^ This feature distinguishes the DDQC from structurally related but distinct phases, such as the Σ phase.^40^Fig. 1d shows the corresponding intensity map of the static structure factor, revealing the expected twelve-fold rotational symmetry. The peaks are not perfectly aligned due to residual phason strain and the presence of defects; such imperfections can vary across simulations and may be reduced by energy-minimization procedures.

### 2.3 2D hexagonal crystal

We also performed MD simulations on a two-dimensional hexagonal crystal at a fixed density of *ρσ*^2^ = 1.05, using the same pairwise interaction potential as the DDQC, eqn (2). Fig. 1e shows a typical configuration of the hexagonal crystal, along with the corresponding intensity map of the static structure factor in Fig. 1f. According to previously reported phase diagrams,^49,51^ this system exhibits a stable hexagonal crystal at densities *ρσ*^2^ > 1. Initial configurations were generated using the same linear cooling protocol as for the DDQC, followed by equilibration at a fixed final temperature of *k*_B_*T*/*ε* = 0.16, using the same thermostat as for the DDQC. After equilibration, the configurations were minimized using the conjugate gradient algorithm. Due to this procedure, samples invariably contained defects, so we use the same minimized configuration as the starting point for all temperatures, making sure that the defect topology is fixed and only thermal activation changes the dynamics. For all temperatures, the system was initialized in the temperature range *k*_B_*T*/*ε* ∈ [0.16,0.185] and equilibrated for 2.5 × 10^6^*τ*_QC_ (5 × 10^8^ MD time steps), corresponding to approximately 25–40 structural relaxation times *τ*_*α*_ across the investigated temperature range (see Fig. 4e). All reported quantities were averaged over five independent samples.

## 3 Dynamical properties

### 3.1 Mean-squared displacement

The mean-squared displacement (MSD) is a key measure for characterizing single-particle dynamics in condensed-matter systems. In two-dimensional systems, however, direct measurements of dynamical quantities are complicated by long-wavelength thermal fluctuations in particle positions, as described by the Mermin-Wagner theorem.^53^ These fluctuations can obscure true signatures of dynamic arrest or caging. To mitigate this effect, we adopt the cage-relative mean-squared displacement (CR-MSD) approach, as proposed in ref. 38, which filters out collective long-wavelength motions by redefining displacements relative to their local neighborhood. The CR-MSD is computed as3
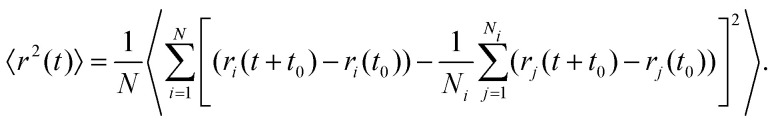


In eqn (3), the second term subtracts the displacement of the center of mass of the local cage surrounding each particle *i*, defined by its *N*_*i*_ nearest neighbors within a cutoff *r*_*c*_. We use *r*_c_ = 1.4*σ* for the DDQC and hexagonal crystal, and *r*_c_ = 1.2*σ*_L_ for the supercooled liquid.

**Fig. 2:**
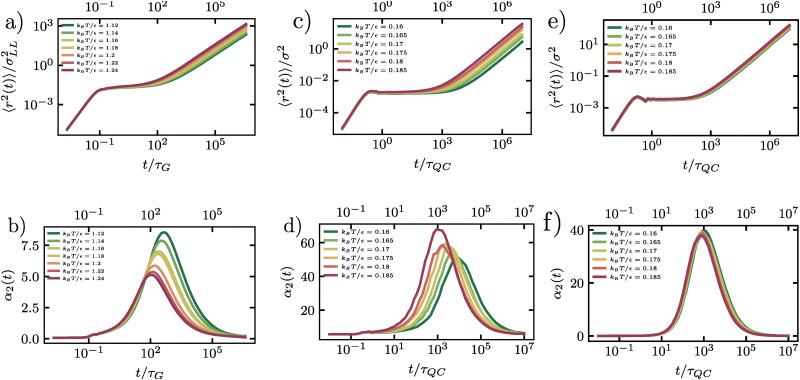
Cage-relative mean-squared displacement (MSD) 〈*r*^2^(*t*)〉 and non-Gaussian parameter *α*_2_(*t*) for (a) and (b) a supercooled liquid, (c) and (d) a dodecagonal quasicrystal, and (e) and (f) a hexagonal crystal. The emergence of a cage-trapping plateau in the MSD indicates transient localization of particles within cages formed by their neighbors, before transitioning to diffusive motion at longer times. Peaks in *α*_2_(*t*) highlight the emergence of dynamical heterogeneity, with characteristic time scales t* associated with cage-breaking events.

We first examine the dynamics of the supercooled binary liquid, shown in Fig. 2a. Following an initial ballistic regime, the system exhibits the characteristic behavior of glass-forming liquids: a pronounced cage-trapping plateau that becomes more extended as temperature decreases. This plateau reflects the transient localization of particles within cages formed by their disordered neighbors, a phenomenon well-established in the literature.^31,54^ The subsequent departure from this plateau at longer times marks the onset of structural relaxation, where particles escape their local cages through cooperative rearrangements, eventually leading to diffusive motion. The monotonic decrease in plateau height and the concomitant increase in plateau duration with decreasing temperature are consistent with the standard picture of dynamical slowdown near the glass transition.

Turning to the dodecagonal quasicrystal (DDQC), shown in Fig. 2c, we observe a strikingly similar dynamical behavior despite the presence of quasi-long-range orientational order. Like the supercooled liquid, the DDQC exhibits a pronounced intermediate-time plateau in the MSD, indicative of particle trapping. However, the microscopic origin of this trapping is fundamentally different: it arises from the complex, non-periodic potential-energy landscape imposed by the quasicrystalline tiling, rather than from random crowding. In this regime, particle motion is constrained by the local symmetry, and diffusion proceeds through activated rearrangements that must overcome energy barriers associated with local tile reconfigurations, including phason-like events.^35,37^ The close similarity in both the timescale and shape of the MSD curves for the DDQC and the supercooled liquid suggests that the rugged quasiperiodic potential effectively mimics the frustration present in disordered glasses. We again observe a decrease in plateau height and a corresponding increase in plateau duration as the temperature is lowered.

Finally, the hexagonal crystal, shown in Fig. 2e, serves as a reference for identifying the role of structural disorder. Recall that, for the crystal, we employed a fixed initial configuration with a static population of defects, such that only thermal activation influences the dynamics. We clearly observe that the crystal also exhibits a transient plateau. This plateau indicates that even ordered systems can display transient localization, where the “cage” is formed by surrounding lattice sites rather than the soft, disordered coordination shells typical of a supercooled liquid. At long times, the mean-squared displacements enter a diffusive regime, facilitated by the pre-existing defect network.

Comparing the three systems reveals a macroscopic universality in their short-time dynamics: all three systems exhibit a cage-trapping plateau indicative of transient localization. However, the origin of these constraints differs between the systems. The supercooled liquid is governed by geometric frustration and continuously evolving disorder; the hexagonal crystal is dominated by energetic lattice constraints and a static defect network; and the DDQC lies between these extremes, possessing long-range order but locally diverse environments that generate a rugged landscape of (free-)energy barriers. This comparison suggests that, although the observation of a plateau seems universal, the underlying mechanism transitions from predominately elastic constraints imposed by the lattice in the crystal to increasingly complex configurational frustration in the quasicrystal and supercooled liquid.

### 3.2 Non-Gaussian parameter

While the mean-squared displacement reveals plateau regimes indicative of transient particle caging, it does not fully characterize the heterogeneous nature of the single-particle dynamics. In dynamical heterogeneous systems, the distribution of single-particle displacements deviates significantly from the Gaussian distribution expected for homogeneous diffusion. To quantify these deviations, we employ the non-Gaussian parameter, *α*_2_(*t*), which measures the kurtosis of the single-particle displacement distribution. For our two-dimensional systems, we evaluate *α*_2_(*t*) using the cage-relative coordinate system introduced in Section 3.1:4
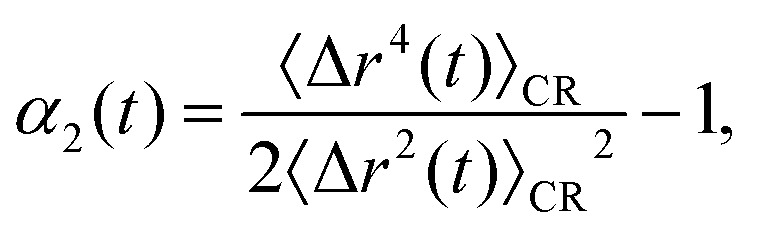
where the brackets denote an ensemble average over all particles and initial times. In the case of purely Gaussian dynamics, *α*_2_(*t*) is zero, while positive values indicate broader-than-Gaussian tails in the displacement distribution, which is a signature of dynamical heterogeneity.

We first consider the supercooled binary liquid, as shown in Fig. 2b. The system exhibits pronounced peaks in *α*_2_(*t*) at intermediate times, confirming the presence of significant dynamical heterogeneity. These peaks correspond to the cage-breaking regime, where the particle population splits into mobile and immobile subgroups. As the temperature decreases, the peak height *α*^max^_2_(t*) increases monotonically, and the characteristic time t* at which this maximum occurs shifts to longer times. This trend indicates that the degree of dynamical heterogeneity grows upon cooling, consistent with increasingly disparate relaxation times among cooperatively rearranging regions as the glass transition is approached.^55^

The dodecagonal quasicrystal (DDQC), presented in Fig. 2d, displays a distinct evolution of dynamical heterogeneity. While the system exhibits pronounced peaks in *α*_2_(*t*), indicating spatially heterogeneous particle mobility, the magnitude of these peaks decreases as the temperature is lowered. This trend contrasts with the behavior of the supercooled liquid and points to a mechanism governed by thermally activated excitations within the quasiperiodic tiling, analogous in spirit to defect-mediated transport in crystals approaching the melting transition.^25^ At high temperatures, sufficient thermal energy is available to activate local particle rearrangements, including phason-like events and other topological changes, leading to a population of mobile particles and pronounced dynamical heterogeneity. As the temperature decreases, these excitations are progressively suppressed, resulting in a more homogeneous single-particle displacement distribution and a reduced non-Gaussian character.

In the hexagonal crystal, shown in Fig. 2f, the behavior of *α*_2_(*t*) similarly diverges from that of the supercooled liquid. Here, the peaks in *α*_2_(*t*) reflect defect-mediated transport, such as vacancy hopping or interstitial motion,^56^ consistent with simulations of bulk melting.^25^ Within the temperature range studied here, *k*_B_*T*/*ε* ∈ [0.16,0.185], thermally activated defect-mediated events persist at all sampled state points. Because the defect topology remains largely fixed, the corresponding *α*_2_(*t*) peak height exhibits only weak temperature dependence.

Collectively, these results demonstrate that while dynamical heterogeneity is a universal feature of particle transport across amorphous, quasiperiodic, and crystalline systems, its temperature dependence reveals fundamentally different origins of dynamic arrest. In the supercooled liquid, the increasing peak height upon cooling signals the growth of spatially correlated, cooperatively rearranging regions driven by packing frustration. Conversely, in both the DDQC and the hexagonal crystal, dynamical heterogeneity is governed by the statistics of localized excitations. In these ordered systems, the non-Gaussian behavior arises from the presence of thermally activated defects, such as phasons or lattice vacancies, placing the quasicrystal dynamically closer to the crystalline state, where heterogeneity diminishes as the available thermal energy is reduced.

**Fig. 3:**
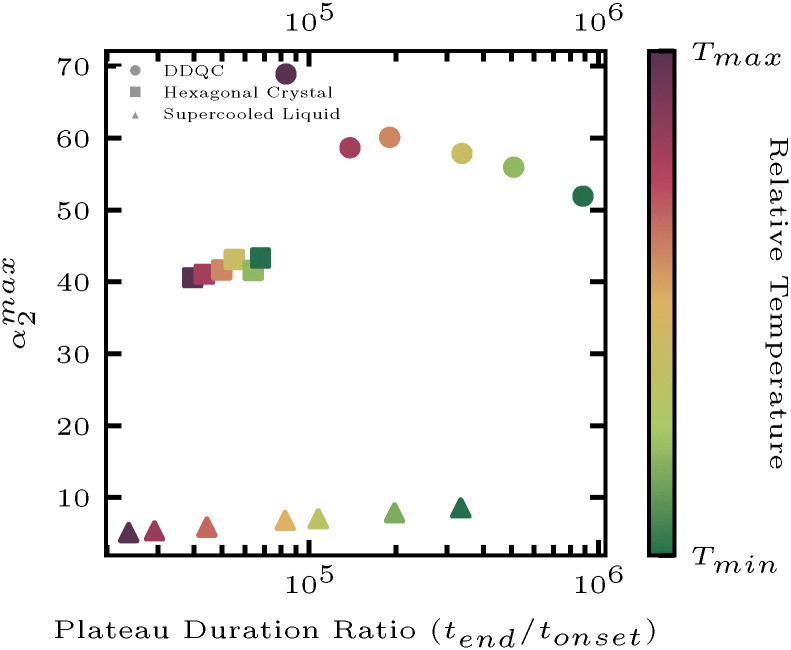
The maximum value of the non-Gaussian parameter *α*^max^_2_*versus* the plateau duration ratio *t*_end_/*t*_onset_, which quantifies the temporal extent of particle caging. Data points are shown for the dodecagonal quasicrystal (circles), hexagonal crystal (squares), and supercooled liquid (triangles), with the color gradient representing the relative temperature from *T*_min_ (green) to *T*_max_ (purple). The supercooled liquid exhibits plateau durations that increase by orders of magnitude upon cooling, while the dynamical heterogeneity remains low (*α*^max^_2_ < 10), indicative of homogeneous, collective cage-breaking processes involving many particles. In contrast, both the DDQC and the hexagonal crystal display significantly higher heterogeneity (*α*^max^_2_ > 40), characteristic of sparse, intermittent transport mechanisms such as vacancy hopping or phason-flip-like rearrangements. Notably, for the DDQC, *α*^max^_2_ decreases as the plateau duration lengthens (decreasing temperature), consistent with the freezing out of thermally activated defects.

Finally, to elucidate the relationship between cage stability and dynamical heterogeneity, we examine the correlation between the duration of the MSD plateau, quantified by the ratio *t*_end_/*t*_onset_, and the maximum non-Gaussian parameter *α*^max^_2_. Fig. 3 reveals a striking decoupling in the supercooled liquid: while the plateau duration extends over two orders of magnitude with decreasing temperature, the dynamical heterogeneity in the single-particle dynamics remains remarkably low and nearly constant (*α*^max^_2_ < 10). We note that *α*_2_(*t*) is computed in cage-relative coordinates, which suppress long-wavelength fluctuations and therefore reduce the apparent non-Gaussianity in the liquid dynamics compared to defect-hopping transport in solids.

These observations suggest that, despite the pronounced structural slowdown, cage breaking in the supercooled liquid is a collective process involving many particles, which maintains a relatively homogeneous single-particle displacement distribution. In sharp contrast, both the DDQC and the hexagonal crystal exhibit much stronger dynamical heterogeneity in the single-particle dynamics (*α*^max^_2_ > 40). This behavior is consistent with transport dominated by rare, intermittent, activated particle rearrangements such as phason-like events, vacancy hopping, and interstitial motion, where mobility is confined to a small subset of particles. Notably, the DDQC exhibits stronger dynamical heterogeneity in the single-particle dynamics (*α*^max^_2_ ∼ 60) than for the hexagonal lattice (*α*^max^_2_ ∼ 40), and also displays a significant longer plateau duration, indicating that the quasiperiodic energy landscape enforces more effective local trapping than the periodic lattice, even though diffusion in both systems proceeds *via* hopping processes. Furthermore, for the DDQC, *α*^max^_2_ decreases as the plateau duration lengthens with decreasing temperature, consistent with the freezing out of thermally activated defects.

### 3.3 Overlap function and dynamical susceptibility

To quantify the temporal relaxation of the structural configurations, we compute the overlap function, *q*(*t*), which measures the fraction of particles that have not undergone significant displacement over a time interval *t*. Consistent with our previous analyses, we employ a cage-relative definition to filter out long-wavelength fluctuations. The overlap function is defined as5
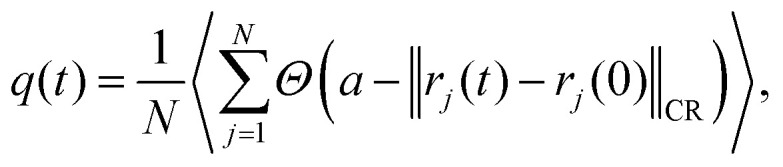
where *Θ* is the Heaviside step function, *a* is a threshold parameter chosen to be smaller than the typical interparticle distance, and 
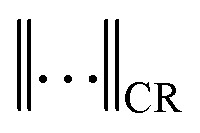
 denotes the magnitude of the cage-relative displacement vector defined in eqn (3). We set *a* = 0.3*σ*_LL_ for the supercooled liquid and *a* = 0.3*σ* for both the quasicrystal and hexagonal crystal. Fluctuations of this order parameter provide a measure of the size of dynamically correlated regions. We quantify these fluctuations using the dynamical susceptibility, *χ*_4_(*t*), defined as^55^

6 *χ*_4_(*t*) = *N*(〈*q*(*t*)^2^〉 − 〈*q*(*t*)〉^2^).

The dynamical susceptibility probes spatial correlations in the dynamics, measuring the extent to which particles move (or remain immobile) cooperatively, and thereby quantifying the size of dynamically correlated regions. This extensive quantity peaks at the timescale of maximal dynamical heterogeneity, with its magnitude proportional to the number of particles involved in cooperative rearrangements.

**Fig. 4:**
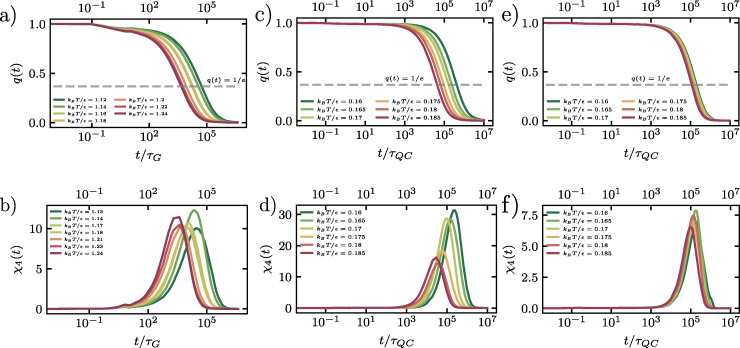
Overlap function *q*(*t*) and dynamical susceptibility *χ*_4_(*t*) for (a) and (b) a supercooled liquid, (c) and (d) a dodecagonal quasicrystal, and (e) and (f) a hexagonal crystal. For the supercooled liquid, *q*(*t*) exhibits a clear two-step relaxation, reflecting transient caging followed by structural relaxation, accompanied by a pronounced peak in *χ*_4_(*t*) that signals strong dynamical heterogeneity. In contrast, the DDQC and the hexagonal crystal relax more smoothly and do not display the two-step decay of *q*(*t*) seen in the supercooled liquid. Remarkably, in the DDQC, *χ*_4_(*t*) increases at the relaxation timescale upon cooling, showing that collective mobility fluctuations persist despite a decrease in *α*_2_(*t*), corresponding with a suppression of large single-particle displacements.

In the supercooled binary liquid, shown in Fig. 4a, the overlap function decays in a two-step process characteristic of glassy dynamics. A short-time plateau reflects particle caging, followed by a stretched-exponential decay as the system relaxes. The corresponding dynamical susceptibility *χ*_4_(*t*) in Fig. 4b exhibits a well-defined peak that coincides with the structural relaxation time of the system. Across the sampled temperatures, the *χ*_4_(*t*) peak height shows an overall tendency to increase upon cooling, although the trend is not strictly monotonic for all neighbouring state points. The largest peak occurs at the lowest temperature studied, reaching a value of approximately 15. This increase reflects an increasing dynamical correlation length and increasingly cooperative structural relaxation as the glass transition is approached.

The dodecagonal quasicrystal (Fig. 4c and d) exhibits a relaxation behavior that is qualitatively similar to the supercooled liquid but without a two-step decay process. While the overlap function decays in a comparable way, the dynamical susceptibility *χ*_4_(*t*) reveals a maximum peak of roughly 30, at least three times more than in the supercooled liquid. This increase indicates that dynamical fluctuations in the quasicrystal involve mobility patterns that are correlated over much larger regions than in the supercooled liquid. Even more strikingly, the dynamical susceptibility *χ*_4_(*t*) increases substantially upon cooling, while *α*_2_(*t*) decreases as shown in Fig. 2d. Importantly, *χ*_4_(*t*) probes fluctuations of the mobility field, whereas the non-Gaussian parameter *α*_2_(*t*) reflects the weight of the single-particle displacement tails. Thus, the large *χ*_4_(*t*) observed in the DDQC does not indicate more heterogeneous single-particle displacements at lower temperatures; instead, it shows that the remaining mobile particles are organized into larger, spatially correlated regions. This decoupling between single-particle intermittency and many-body correlations is examined in more detail in Section 4.

For the hexagonal crystal in Fig. 4e and f, the dynamics is governed by the underlying lattice periodicity and the static defect network set by our initialization protocol. The overlap function decays sharply, and the corresponding *χ*_4_(*t*) maximum peaks are slightly lower than those of the supercooled liquid. Unlike in the supercooled liquid and the DDQC, the peak height remains approximately constant across the temperature range, with only a slight increase at lower temperatures, indicating that the length scale of dynamical correlations is determined by the static defect topology rather than by thermal fluctuations. Thus, the crystal can exhibit pronounced single-particle intermittency without developing the larger, spatially correlated mobility clusters seen in the DDQC. In this sense, *α*_2_(*t*) and *χ*_4_(*t*) do not necessarily track one another across the three systems, as they probe distinct aspects of the dynamics.

Comparing the three systems reveals a sharp distinction in the nature of their dynamical correlations. While the supercooled liquid exhibits a relatively small *α*_2_(*t*), reflecting fairly homogeneous single-particle dynamics, both the DDQC and the hexagonal crystal display pronounced heterogeneous dynamics. In contrast, the dynamical susceptibility *χ*_4_(*t*) is most pronounced in the quasicrystal compared to the hexagonal crystal and the supercooled liquid. The DDQC is particularly striking because its *α*_2_(*t*) decreases upon cooling while *χ*_4_(*t*) simultaneously increases. This decoupling between single-particle intermittency and many-body dynamical correlations cannot be explained solely by a suppression of large single-particle displacements; the spatial organization of the mobility field must also be taken into account.

## 4 Microscopic origin of the dynamical decoupling in DDQC

### 4.1 Cage-relative displacement statistics

**Fig. 5:**
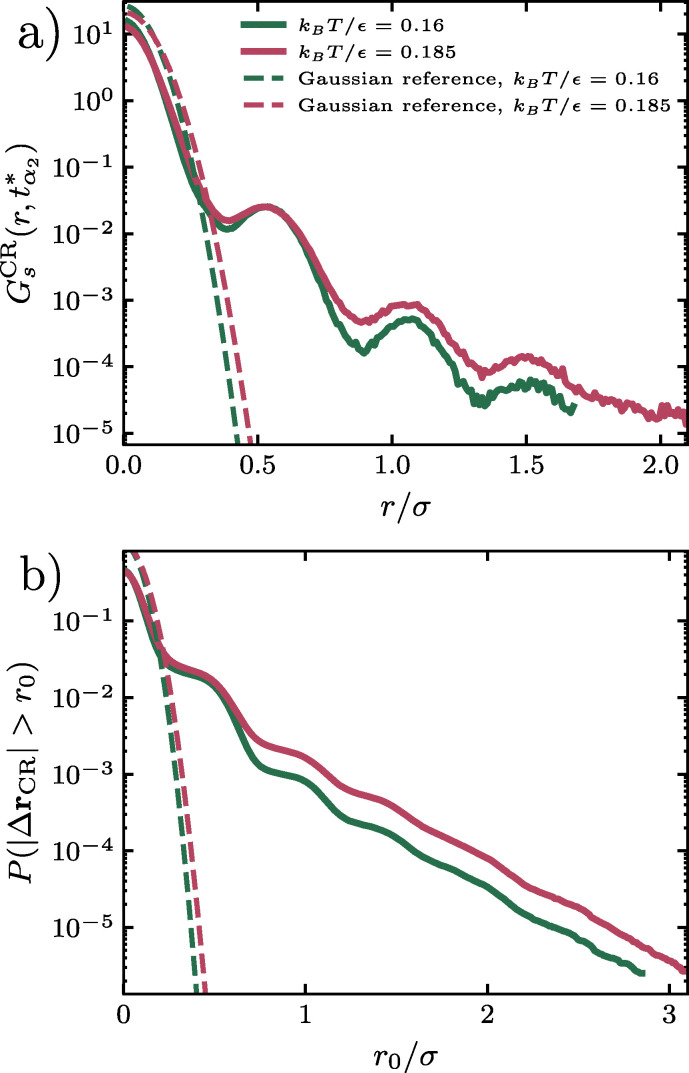
Cage-relative displacement statistics for the dodecagonal quasicrystal at the characteristic time 
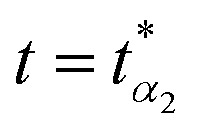
. (a) Area-normalized cage-relative self part of the van Hove function 
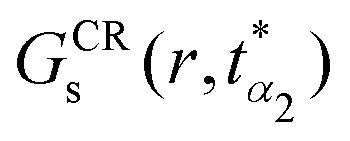
 for *k*_B_*T*/*ε* = 0.16 and 0.185. (b) Corresponding cumulative tail probability *P*(|Δ*r*_CR_| > *r*_0_) as a function of the displacement threshold *r*_0_. The high-temperature state exhibits systematically stronger intermediate- and large-displacement tails, indicating that the decrease of *α*_2_^max^ upon cooling is associated with a suppression of large local rearrangements. In both panels, the temperature-matched Gaussian reference curves decay much more rapidly than the measured distributions, highlighting the pronounced non-Gaussian enhancement of the intermediate- and large-displacement tails at both temperatures.

In Section 3, we identified an unusual dynamical behavior in the DDQC: upon entering the slow-dynamics regime, the non-Gaussian parameter *α*_2_(*t*) decreases, while the dynamical susceptibility *χ*_4_(*t*) increases upon cooling. To clarify the origin of this DDQC-specific decrease in *α*_2_(*t*), we take a closer look at the motion of the particles by measuring the self part of the cage relative van Hove function^57^7
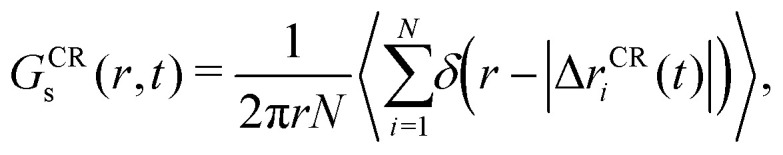
which measures the probability function of cage-relative displacement magnitudes at lag time *t*, averaged over all particles and time origins in two dimensions. Here, *δ* denotes the Dirac delta function. For freely diffusing particles, *G*^CR^_s_(*r*,*t*) would take a Gaussian form in *r*. In Fig. 5a, we plot *G*^CR^_s_(*r*,*t*) evaluated at the characteristic time 
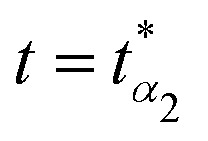
, where the non-Gaussian parameter reaches its maximum, for the DDQC at the highest and lowest temperatures studied, *k*_B_*T*/*ε* = 0.185 and *k*_B_*T*/*ε* = 0.16. In both cases, distinct peaks appear at well-defined positions associated with the quasiperiodic structure of the DDQC, reflecting particles rattling within their local cages before escaping and jumping to neighbouring sites. We further observe that the cage-relative displacement statistics are strongly non-Gaussian, with a dominant near-immobile population and a structured tail extending to larger displacements. Upon cooling, however, the tails at intermediate and large displacements are systematically suppressed. This trend is evident in both 
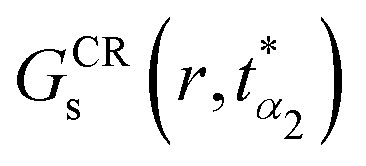
 and the cumulative tail probability8
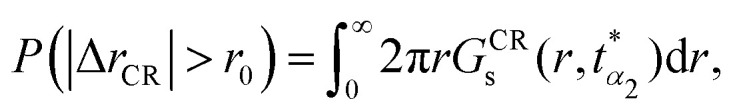
which quantifies the fraction of particles that move farther than a threshold *r*_0_ relative to their local cage, as shown in Fig. 5b. The fraction of mobile particles exceeding the thresholds *r*_0_ = 0.3*σ* and 0.4*σ* is slightly but systematically suppressed upon cooling: *P*(|Δ*r*_CR_| > 0.3*σ*) decreases from 2.62 × 10^−2^ at *k*_B_*T*/*ε* = 0.185 to 2.25 × 10^−2^ at *k*_B_*T*/*ε* = 0.16, while *P*(|Δ*r*_CR_| > 0.4*σ*) decreases from 2.17 × 10^−2^ to 1.91 × 10^−2^. In line with this trend, the 90th and 95th percentiles of |Δ*r*_CR_| shift modestly from approximately 0.145*σ* and 0.195*σ* at *k*_B_*T*/*ε* = 0.185 to 0.125*σ* and 0.175*σ* at *k*_B_*T*/*ε* = 0.16. The high-temperature state retains a larger tail probability across this range, indicating that the reduction of *α*^max^_2_ at low temperature reflects a genuine decrease in the population of particles undergoing large displacements. The next subsection demonstrates that the particles that remain mobile are nevertheless organized into strongly correlated clusters.

### 4.2 Time-resolved mobile-cluster size distribution

The self part of the van Hove function shows that large particles displacements are suppressed upon cooling, but it does not reveal whether the remaining mobile particles are spatially correlated. To probe these collective spatial correlations, we analyze cage-relative displacements over a lag time *t* measured from a time origin *t*_0_. We focus on lag times relative to 
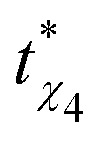
, defined for each temperature as the time at which the dynamical susceptibility *χ*_4_(*t*) reaches its maximum. We then define the cage-relative mobility field

9 *μ*_*i*_(*t*_0_,*t*) = |Δ*r*_*i*,CR_(*t*_0_,*t*)|

and identify mobile particles using

10 *m*_*i*_ = *Θ*(*μ*_*i*_ − *a*),

with threshold *a* = 0.3*σ*.

**Fig. 6:**
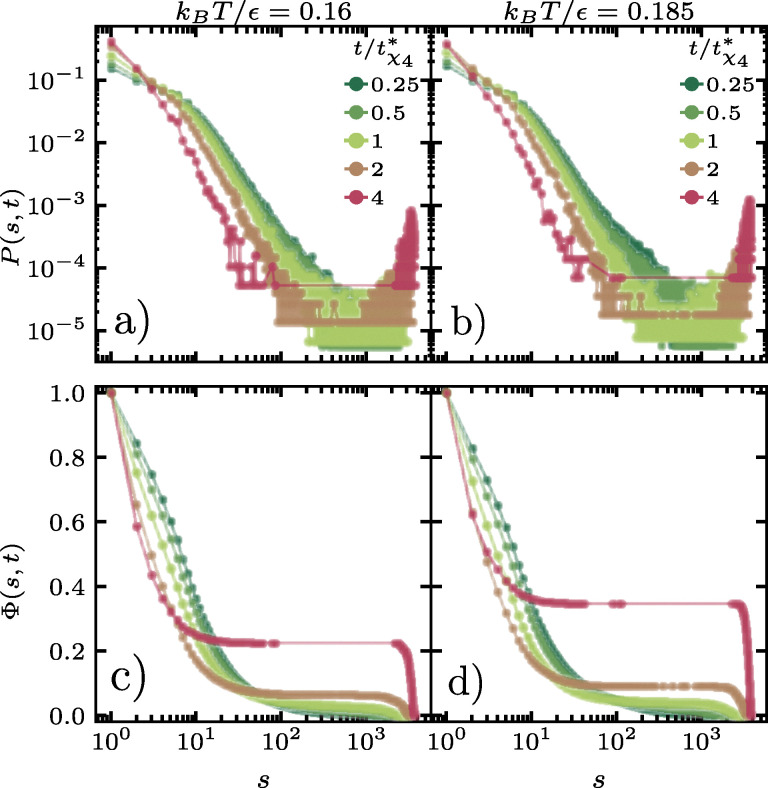
Time-resolved mobile-cluster growth in the dodecagonal quasicrystal for threshold *a* = 0.3*σ*. Panels (a) and (c) correspond to *k*_B_*T*/*ε* = 0.16, while panels (b) and (d) correspond to *k*_B_*T*/*ε* = 0.185. The top row shows the mobile-cluster-size distribution *P*(*s*,*t*), and the bottom row shows the cumulative mobile-cluster-size distribution 
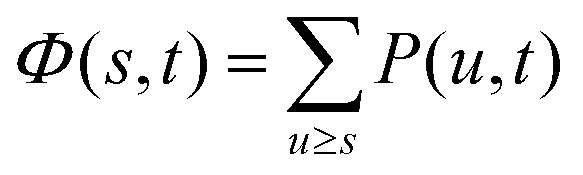
. At both temperatures, increasing 
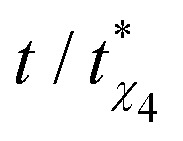
 increases the fraction of clusters exceeding a given size *s*.

Two particles identified as mobile are assigned to the same cluster when their periodic minimum-image separation in the configuration at time origin *t*_0_ is smaller than 1.4*σ*. The cluster size *s* is defined as the number of mobile particles belonging to the same cluster. From these clusters we construct the time-resolved cluster-size distribution *P*(*s*,*t*), defined as the probability that a mobile cluster observed at lag time *t* has size *s*. Fig. 6 summarizes the resulting DDQC cluster-growth data at relative times 
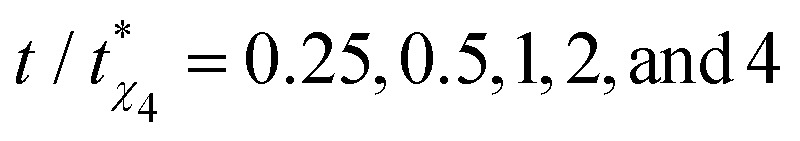
. Panels Fig. 6a and b show the mobile-cluster-size distribution *P*(*s*,*t*), while panels Fig. 6c and d display the cumulative mobile-cluster-size distribution11
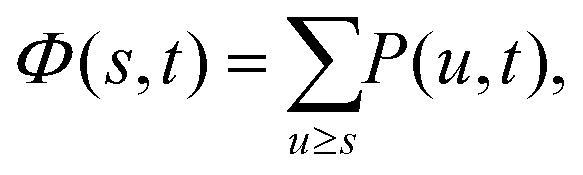
representing the fraction of mobile clusters with size at least *s*. At both temperatures, increasing 
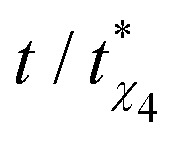
 makes large cluster sizes more probable and small and intermediate cluster sizes less likely; in *Φ*(*s*,*t*) this appears as a larger fraction of clusters above a given size. The temperature dependence is weak at 
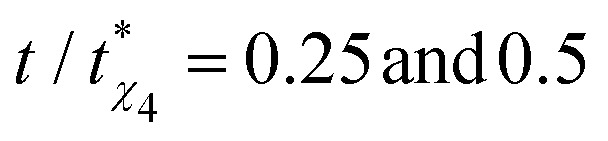
, where the *k*_B_*T*/*ε* = 0.16 and 0.185 curves remain close and partly overlap. By 
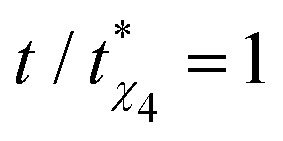
, and even more clearly at 2 and 4, large cluster sizes are more likely at *k*_B_*T*/*ε*= 0.185, while small and intermediate cluster sizes are dominant at *k*_B_*T*/*ε*= 0.16. At matched 
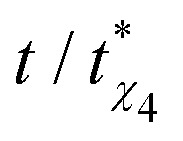
, the cluster-size distributions therefore show that large connected mobile clusters are less probable at *k*_B_*T*/*ε* = 0.16 than at 0.185, while small and intermediate clusters are more probable.

### 4.3 Cluster morphology

**Fig. 7:**
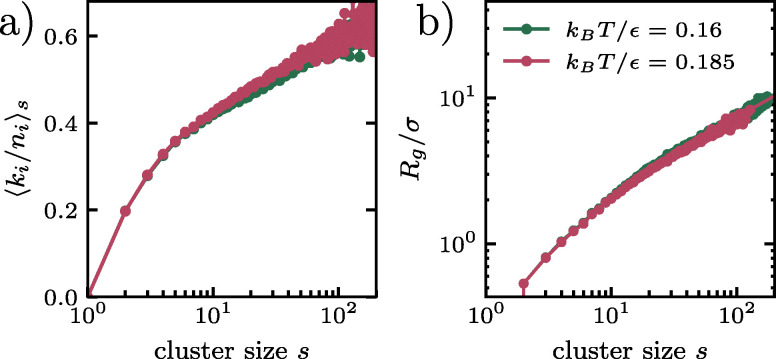
Morphology of mobile clusters in the dodecagonal quasicrystal at 
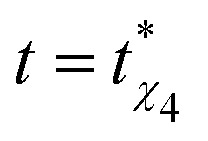
 for a threshold value *a* = 0.3. (a) Cluster compactness 〈*k*_*i*_/*n*_*i*_〉_*s*_ as a function of cluster size *s*. (b) Radius of gyration *R*_g_(*s*) as a function of cluster size *s* on log–log axes. Both the cluster compactness 〈*k*_*i*_/*n*_*i*_〉_*s*_ and the radius of gyration *R*_g_(*s*) increases with cluster size, while both curves for *k*_B_*T*/*ε* = 0.16 and 0.185 are very similar. The visible curvature of *R*_g_(*s*) on log–log axes indicates that the observed clusters are not described by a single power law over the full size range.

To determine whether cooling affects only the extent of the cluster size distribution or also the morphology of the clusters, we characterize the geometry of the mobile clusters using two complementary observables. The first is the internal connectivity of each particle, defined as12
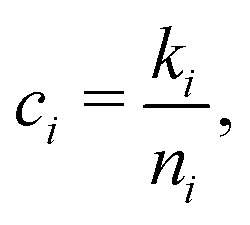
where *n*_*i*_ is the number of geometric neighbors of particle *i* within 1.4*σ* at the time origin, and *k*_*i*_ is the number of those neighbors that are mobile and belong to the same mobile cluster. The cluster compactness is then reported as 〈*k*_*i*_/*n*_*i*_〉_*s*_, averaged over all particles belonging to clusters of size *s*. As a second, more global morphology parameter, we measure the radius of gyration *R*_g_(*s*) of the clusters. Fig. 7 shows that cluster compactness increases steadily with cluster size *s* at both temperatures. For the main threshold *a* = 0.3, the regime-averaged compactness rises by roughly 0.20 between the small-cluster regime 1 ≤ *s* ≤ 8 and the intermediate regime 10 ≤ *s* ≤ 80 at both temperatures, *i.e.*, from about 0.29 to about 0.49 in each case. This size-driven increase is much larger than the residual temperature difference at fixed regime. Larger clusters therefore occupy a substantially larger fraction of the local quasicrystal neighbor shell than small clusters, *i.e.*, they are more internally connected and less sparse. In contrast, the two temperature curves remain very close at fixed *s*, indicating that the internal filling of a cluster is determined primarily by its size and only weakly by temperature.

The radius of gyration provides an independent confirmation of the same conclusion. It grows monotonically with cluster size and remains very similar at fixed *s* for *k*_*B*_*T*/*ε* = 0.16 and 0.185, indicating that the overall spatial extent of a cluster is only weakly temperature dependent once the cluster size is fixed. At the same time, the visible curvature of *R*_g_(*s*) on log–log axes shows that the observed clusters are not described by a single self-similar power law over the full size range resolved here. This observation is consistent with the compactness results: cluster geometry evolves with size, from relatively sparse small clusters to more internally connected intermediate clusters, but does so in essentially the same way at both temperatures. Thus, the main temperature dependence is not a qualitative change in cluster shape, but rather the extent to which the cluster-size distribution coarsens. We verified that these qualitative trends, the size-driven increase in compactness and the weak temperature dependence at fixed cluster size, are preserved when the mobility threshold is raised to *a* = 0.4*σ*.

## 5 Vibrational properties

### 5.1 Vibrational density of states

We next investigate how structural order manifests in the vibrational spectrum. To analyze these properties, we compute the dynamical matrix for each system, defined as^30^13
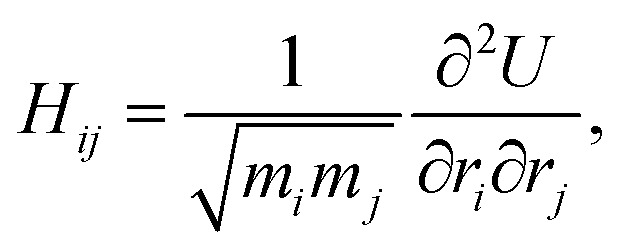
where *r*_*i*_ represents the spatial coordinate of particle *i*, and *m*_*i*_ denotes its mass. The total potential energy of the system is given by 
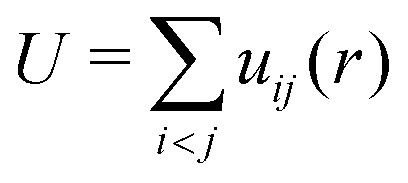
, where *u*_*ij*_(*r*) denotes the interaction potential between particles *i* and *j*.

For all systems, we compute the dynamical matrix *H*_*ij*_ at energy-minimized configurations. These configurations were first equilibrated at specific parent temperatures: *k*_B_*T*/*ε* = 0.16 for the DDQC and the hexagonal crystal, and *k*_B_*T*/*ε* = 1.12 for the supercooled liquid. Energy minimization is performed using the Fast Inertial Relaxation Engine (FIRE) algorithm,^58^ which effectively quenches the system to zero temperature, ensuring that it resides at a local minimum of its respective potential-energy surface. The choice of parent temperature is crucial because it determines which region of the energy landscape the system explores prior to minimization. Once the Hessian matrices are constructed, we diagonalize them to obtain the full spectrum of eigenvalues and eigenvectors.

From the eigenvalues *λ*_*l*_, with *l* = 1, 2, …, 2*N* and *N* the total number of particles, we obtain the corresponding normal mode frequencies as 
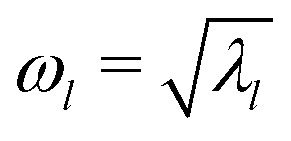
. Using these frequencies, we compute the vibrational density of states, *D*(*ω*), defined as14
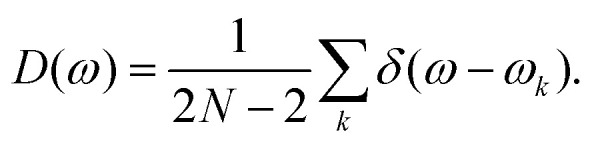


The vibrational density of states (vDOS) counts the number of vibrational modes at each frequency and forms the basis for calculating various thermodynamic properties, such as the heat capacity.^59,60^ In practice, we compute the vDOS by binning the frequencies into uniform intervals, counting the number of modes in each bin, and normalizing by the total number of modes and the bin width.

**Fig. 8:**
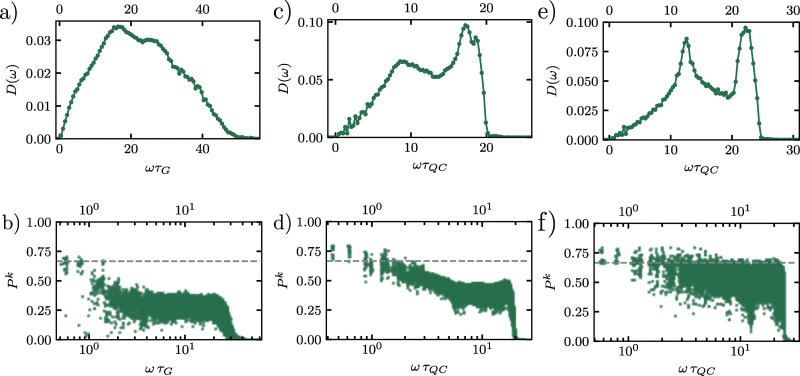
Vibrational density of states *D*(*ω*) as a function of frequency *ω* for (a) a supercooled liquid, (c) a dodecagonal quasicrystal, and (e) a hexagonal crystal. In panels (b), (d) and (f) we show the participation ratio *P*^*k*^ as a function of frequency *ω* for the supercooled liquid, the dodecagonal quasicrystal and the hexagonal crystal, respectively. The plane-wave limit *P*^*k*^ = 2/3 is shown as a dotted line in the panels.

Fig. 8a displays the vDOS, *D*(*ω*), for the supercooled liquid. As in the other systems, the two lowest-frequency modes correspond to the Goldstone modes, which are effectively zero within numerical accuracy. Beyond these modes, the low-frequency spectrum scales linearly with frequency, *i.e.*, *D*(*ω*) ∝ *ω*, consistent with the Debye model for two-dimensional systems and reflecting the dominance of long-wavelength acoustic phonons.^61^ At intermediate frequencies (*ωτ*_G_ ≈ 10), the liquid deviates from Debye behavior, displaying a broad peak known as the boson peak,^62^ indicative of an excess of vibrational modes and a hallmark of disordered solids.^63^ At higher frequencies, the vDOS gradually decreases, signaling a transition from extended, acoustic-like modes to increasingly localized vibrational excitations. Next, we examine the vDOS for the DDQC, presented in Fig. 8c. Similar to the supercooled liquid, the DDQC exhibits linear Debye scaling (*D*(*ω*) ∝ *ω*) at low frequencies, confirming the presence of acoustic phonon modes despite the absence of translational periodicity. At intermediate and high frequencies, however, its behavior differs markedly from the amorphous case. Instead of a smooth boson peak, the DDQC displays pronounced peaks at *ωτ*_QC_ ≈ 8, along with additional features at higher frequencies. These peaks likely arise from phason dynamics, collective rearrangements inherent to quasiperiodic order, and from the presence of pseudo-Brillouin zone boundaries, which generate gaps and pile-ups in the density of states, analogous to observations in experimental studies of icosahedral quasicrystals.^64,65^

Finally, we analyze the hexagonal crystal, shown in Fig. 8e. At low frequencies, the spectrum exhibits the same universal linear Debye scaling observed in the supercooled liquid and DDQC, confirming the presence of acoustic phonons. At high frequencies, however, the behavior differs sharply: the crystalline vDOS exhibits pronounced peaks corresponding to van Hove singularities, where the phonon group velocity vanishes. This vanishing reflects the breakdown of coarse-grained continuum linear elasticity at wavelengths comparable to the lattice spacing. The lattice's translational periodicity renders these features especially sharp, because it produces well-defined Brillouin-zone boundaries that pin the singularities to specific frequencies; by contrast, the DDQC supports only pseudo-Brillouin zones, broadening the analogous features visible at *ωτ*_QC_ ≈ 8. These features are absent in the supercooled liquid, which lacks long-range order, and are significantly sharper than the broader peaks observed in the DDQC. Thus, while dynamical observables such as the MSD and non-Gaussian parameter reveal universal aspects of particle motion across all three systems, the vDOS serves as a distinct structural fingerprint, clearly distinguishing the perfect translational periodicity of the hexagonal crystal from the quasiperiodic order of the DDQC and the amorphous nature of the supercooled liquid.

### 5.2 Participation ratio

We now analyze the spatial character of the normal modes by examining the participation ratio (PR), which quantifies the extent to which vibrational modes are localized or delocalized across the system. The PR for each vibrational mode *k* is defined as15
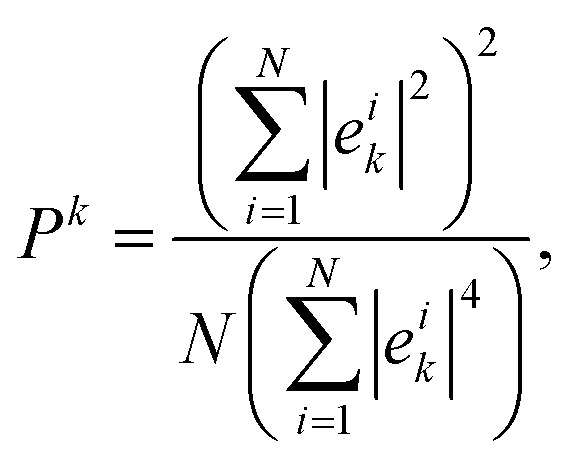
where *e*_*k*_^*i*^ represents the eigenvector component for particle *i* in mode *k*. In two-dimensional systems, the participation ratio reaches *P*^*k*^ = 2/3 for perfectly extended plane-wave modes, while it approaches *P*^*k*^ ≈ 1/*N* for highly localized modes.^66^ Thus, the PR provides a measure of the effective fraction of particles that contribute significantly to a given vibrational mode, allowing us to distinguish extended, collective vibrations from modes that are spatially confined to small regions of the system.

Fig. 8b presents the PR spectrum for the supercooled liquid, calculated from the energy-minimized configuration at a parent temperature of *k*_B_*T*/*ε* = 1.12. At low frequencies (*ωτ*_G_ < 1), the PR approaches approximately 0.60, slightly below the theoretical plane-wave limit of *P*^*k*^ = 2/3, which reflects the system's intrinsic structural disorder that persists even in the most extended vibrational modes. As the frequency increases into the intermediate range (1 < *ωτ*_G_ < 10), we observe substantial variability in the PR values, with a standard deviation of roughly 0.15 around *ωτ*_G_ ≈ 5. This indicates that modes at comparable frequencies can differ markedly in their degree of localization. This heterogeneity in vibrational mode localization is reminiscent of the dynamical heterogeneity revealed by the non-Gaussian parameter (Section 3.2), suggesting a potential correlation between vibrational mode localization and the propensity for particle rearrangements. At higher frequencies (*ωτ*_G_ > 10), the *P*^*k*^ values drop below 0.2, signaling the emergence of highly localized excitations confined to small clusters of particles. The binned results (squares with error bars) are calculated using uniformly spaced frequency windows, containing at least 15 modes each. The error bars correspond to one standard deviation, and the observed fluctuations represent genuine variations in mode structure within each frequency band rather than statistical noise.

In Fig. 8d, we plot the PR spectrum as a function of frequency *ωτ*_QC_ for the DDQC, calculated from the energy-minimized configuration at a parent temperature of *k*_B_*T*/*ε* = 0.16. At low frequencies (*ωτ*_QC_ < 2), the PR approaches the theoretical plane-wave limit of *P*^*k*^ = 2/3, indicating that these modes are extended and phonon-like. As the frequency increases into the intermediate regime (2 < *ωτ*_QC_ < 20), the *P*^*k*^ values gradually decrease, reflecting the progressive localization of the vibrational modes. At higher frequencies (*ω* > 20), the *P*^*k*^ drops sharply toward zero, signaling the emergence of highly localized excitations.

The PR spectrum of the DDQC reveals both similarities and notable differences compared to the supercooled liquid. Both systems exhibit the expected decrease in *P*^*k*^ with increasing frequency *ω*, but the DDQC maintains *P*^*k*^ values much closer to the plane-wave limit (∼0.65) over a broader low-frequency range (*ωτ*_QC_ < 2) than the supercooled liquid (*ωτ*_G_ < 1). This systematic offset, amounting to roughly an 8% higher *P*^*k*^ at *ωτ*_QC/G_ = 1.5, reflects the more pronounced structural coherence of the DDQC despite its lack of periodicity. At intermediate frequencies, both systems show pronounced fluctuations in PR, yet the DDQC maintains a slightly higher average *P*^*k*^, indicating that its vibrational modes remain more extended than those of the supercooled liquid. This enhanced collectivity likely arises from the DDQC's quasi-long-range order, which provides more uniform local environments compared to the disordered structure of the supercooled liquid.

Finally, we examine the participation ratio of the hexagonal crystal, energy–minimized from a parent temperature of *k*_B_*T*/*ε* = 0.16, shown in Fig. 8f. Consistent with its long–range translational order, the crystal exhibits the most coherent vibrational character among the three systems. At low frequencies, the values of *P*^*k*^ align precisely with the theoretical plane-wave limit of *P*^*k*^ = 2/3, indicating the presence of ideal, extended phonon modes. This behavior contrasts sharply with the supercooled liquid in Fig. 8b, where *P*^*k*^ drops rapidly, and with the DDQC in Fig. 8d, which shows a more gradual decay in mode extension at intermediate frequencies. A key distinction is that the hexagonal crystal sustains a high participation ratio over a much broader frequency range, remaining close to the extended-mode limit up to *ωτ*_QC_ ≈ 20. Beyond this point, the PR drops abruptly, marking a sharp transition to localized modes. This transition coincides with the high-frequency van Hove singularity visible in the vibrational density of states, reflecting a distinct boundary in the phonon spectrum that is absent in both the disordered and the quasiperiodic system.

These characteristics of the participation ratio provide a vibrational fingerprint for each system. Both the hexagonal crystal and the DDQC sustain extended vibrational modes at intermediate frequencies, consistent with their collective transport mechanisms, which are standard lattice vibrations in both the crystal and quasicrystal as well as phason-related dynamics in the quasicrystal. In contrast, the supercooled liquid exhibits rapid mode localization in this regime, reflecting its inherently heterogeneous and spatially disordered mobility. This vibrational hierarchy places the DDQC closer to the crystalline state in terms of vibrational coherence, despite its glass-like dynamical arrest. The frequency-domain picture thus complements the real-space analysis: the DDQC combines slow caging dynamics with a level of vibrational coherence characteristic of an ordered solid rather than a supercooled liquid.

## 6 Concluding remarks

In this work, we investigated the dynamical behavior and vibrational properties of three structurally distinct two-dimensional systems: a dodecagonal quasicrystal (DDQC), a hexagonal crystal, and a supercooled binary liquid. Despite their differing degrees of structural order, all three systems exhibit transient caging in the mean-squared displacement and clear deviations from Gaussian single-particle diffusion. These findings show that intermittent slow dynamics are not exclusive to disordered systems; quasiperiodic and defect-containing ordered solids can also generate rugged dynamical energy landscapes.

The DDQC nevertheless displays a particularly unusual combination of observables. Upon cooling, the non-Gaussian parameter decreases, and the cage-relative van Hove analysis shows that the large-displacement tails are genuinely suppressed. At the same time, the dynamical susceptibility increases steadily upon cooling. Our real-space cluster analysis resolves this apparent contradiction: upon cooling, fewer particles undergo large displacements, but the remaining mobile particles form extended, spatially correlated clusters. Furthermore, the cluster-size distribution coarsens as the lag time approaches and exceeds 
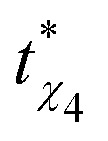
, with stronger coarsening at *k*_B_*T*/*ε* = 0.185 than at 0.16. The compactness of mobile clusters increases mainly with cluster size and only weakly with temperature, indicating that temperature primarily affects the cluster-size distribution rather than altering cluster morphology.

In the frequency domain, the apparent dynamical similarities give way to clear vibrational differences. While all three systems follow the expected Debye scaling at low frequencies, the supercooled liquid develops a boson peak and rapid mode localization, whereas the DDQC maintains a more coherent vibrational response, remaining closer to the hexagonal crystal in its participation-ratio spectrum. Dynamically, the DDQC shows a clear decoupling between single-particle statistics and collective motion. Cooling suppresses large single-particle displacements, while the cluster analysis shows that collective motion persists but is distributed more toward small and intermediate mobile clusters than toward large ones. Vibrationally, it remains substantially closer to an ordered solid than to the supercooled liquid.

A fuller macroscopic characterization of the elastic and viscoelastic response, including elastic moduli of the ordered phases and transport coefficients associated with defect- or phason-mediated relaxation, would be a valuable direction for future work, but lies beyond the scope of the present microscopic study.

## Conflicts of interest

There are no conflicts to declare.

## Appendices

### Appendix A: single-particle trajectories and local dynamics

In this Appendix, we examine how individual particles navigate their local environments, complementing the mean-squared displacement, non-Gaussian parameter, and vibrational analyses in the main text.

#### 1 Single-particle trajectories

**Fig. 9:**
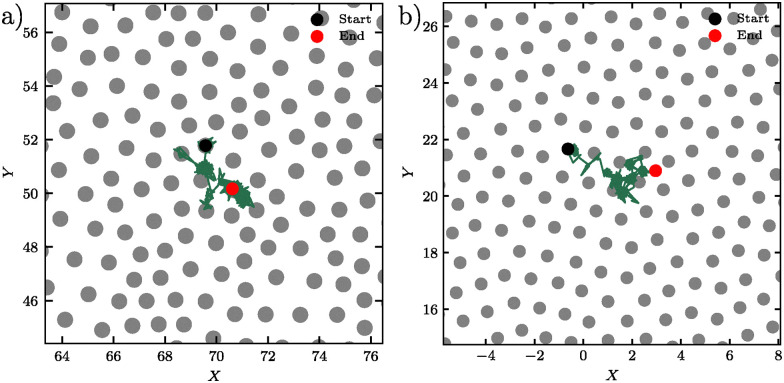
Single-particle trajectories revealing similar intermittent dynamics in structurally distinct systems. Representative trajectories of individual mobile particles tracked over 10^6^ MD time units. Gray circles show neighboring particles in the initial configuration, with the selected particle's initial position marked by a black circle and final position by a red circle. (a) Trajectory of a mobile particle in the supercooled liquid at *k*_B_*T*/*ε* = 0.95, showing characteristic cage rattling followed by discrete jumps. (b) Trajectory of a mobile particle in the dodecagonal quasicrystal at *k*_B_*T*/*ε* = 0.17, exhibiting similar step-like motion despite the system's quasi-long-range order.

Fig. 9 shows representative trajectories of individual particles in both the DDQC and the supercooled liquid over 10^6^ MD time units. We focus on highly mobile particles, as many particles remain effectively immobile, reflecting the dynamical heterogeneity quantified in Section 3.2. In both systems, particle motion proceeds *via* localized vibrations within the cage formed by neighboring particles, followed by sudden jumps, after which particles reside transiently in new metastable positions before the next jump. This intermittent behavior produces trajectories characterized by cage-trapping plateaus of confined motion separated by abrupt jumps.

In the supercooled liquid, shown in Fig. 9a, particle trajectories exhibit this intermittent pattern: extended periods of cage rattling followed by abrupt displacements, indicative of cooperative rearrangements involving multiple neighbors. Although we do not resolve the underlying microscopic mechanisms, these events resemble structural transitions reported in the glass literature—such as T1-like processes^27,68,69^—which involve neighbor exchanges and collective particle motion while preserving local structural correlations.

In the DDQC (Fig. 9b), the discrete jumps resemble phason flips—collective rearrangements that preserve quasicrystalline order while enabling structural relaxation.^35,37^ While our data cannot definitely identify these events, the trajectory characteristics are consistent with the coordinated motion expected in phason-mediated rearrangements, where local tiling configurations are transformed without disrupting the overall dodecagonal symmetry. Previous studies have shown that these processes are governed by well-defined activation energies,^36^ providing a plausible explanation for the thermally activated diffusion in QCs and the temperature dependence of the non-Gaussian parameter shown in Fig. 2d.

The similarity in single-particle trajectories provides a microscopic basis for the comparable features observed in the mean-squared displacement (Section 3.1) and non-Gaussian parameters (Section 3.2) analyses. In both systems, the cage-trapping plateaus in the MSD reflect particle trapping within transient cages formed by neighboring particles, while subsequent cage-breaking events lead to diffusive behavior at longer time scales. The spatial and temporal heterogeneity of these cage-breaking events arising from the aperiodic lattice give rise to pronounced peaks in the non-Gaussian parameters.

These observations connect the dynamical analysis of Section 3 with the vibrational properties discussed in Section 5. Although both systems display similar caging and non-Gaussian behavior, the underlying mechanisms differ fundamentally: in the DDQC, rearrangements are governed by phason dynamics within a quasiperiodic lattice, whereas in the supercooled liquid they arise from cooperative motion in a disordered energy landscape.

The vibrational analysis in the next Appendix section provides a complementary characterization of the eigenmodes, but is not used as primary evidence for the microscopic transport mechanism. The single-particle trajectory analysis thus completes the comparative picture by linking the macroscopic similarities in dynamical heterogeneity to microscopic differences in structural and vibrational behavior.

#### 2 Representative real-space mobility maps

**Fig. 10:**
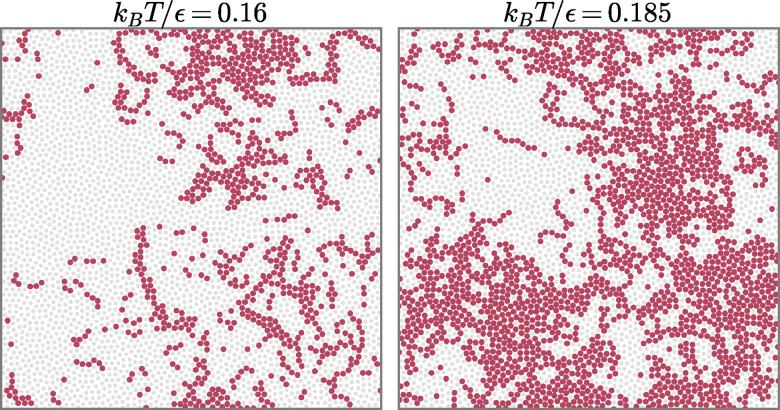
Representative real-space mobility maps in the DDQC at 
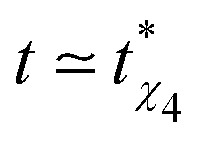
. Gray points show all particles and red points show mobile particles defined by the cage-relative threshold *a* = 0.4*σ*, used here for visual clarity. In each panel, mobility is determined using eqn (10) from a representative pair of configurations obtained from the same replica, consisting of an initial configuration at time *t*_0_ and a second snapshot at time *t*_0_ + Δ*t*, with 
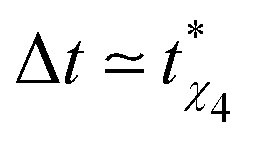
. However, the particle coordinates are those of the initial configuration at time *t*_0_. The low-temperature state at *k*_B_*T*/*ε*= 0.16 is shown on the left and the high-temperature state at *k*_B_*T*/*ε*= 0.185 on the right.

To complement the single-particle trajectories, Fig. 10 presents illustrative maps of the mobility field in the DDQC at 
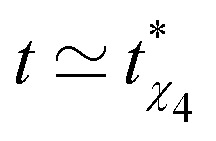
 for representative snapshot pairs, constructed from the cage-relative mobility field and mobile-particle indicator defined in eqn (9) and (10). We use the higher threshold *a* = 0.4*σ* to emphasize the most mobile regions while remaining within the robustness range already analyzed in the main text.

At both temperatures, mobile particles form extended, contiguous patches rather than being uniformly distributed throughout the sample. The higher-temperature state shows broader, more connected mobile regions, while the lower-temperature state remains spatially clustered but less coarsened. These representative maps are consistent with the quantitative analysis in Section 4: cooling reduces the extent of collective mobile regions without eliminating the spatial organization of the mobility field.

### Appendix B: Topological singularities in vibrational phase fields

Here, we provide an auxiliary analysis of the phase fields associated with the vibrational eigenmodes. For each eigenmode, we construct a continuous phase field *θ*(*r*) from the eigenvector components and compute the winding number *q* around elementary cells. Cells with *q* = ±1 are identified as phase singularities of the eigenfield. In what follows, we use the term defect only as a shorthand for these singularities in the constructed phase field, not as structural defects in the particle configuration.

To compare the three systems on equal footing, we plot the singularity density as a function of the reduced frequency *ω*/*ω*_D_, where *ω*_D_ is the Debye frequency.

**Fig. 11:**
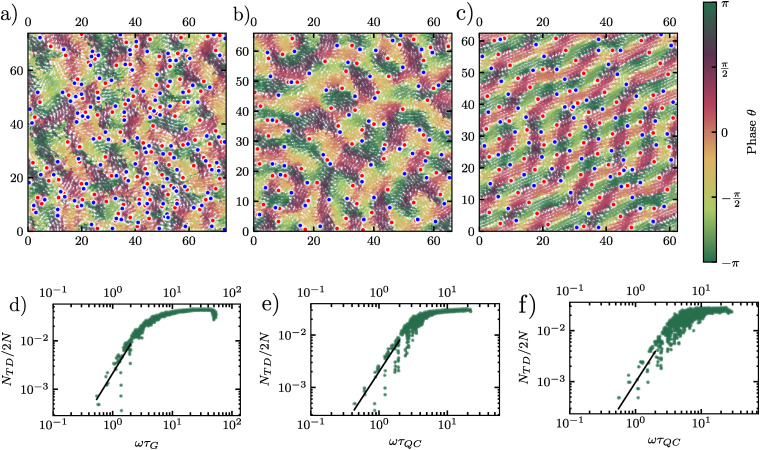
Top panels: representative phase fields constructed from vibrational eigenmodes for (a) a supercooled liquid at frequency *ωτ*_G_ = 4.29, (b) a dodecagonal quasicrystal at *ωτ*_QC_ = 3.08, and (c) a hexagonal crystal at *ωτ*_QC_ = 4.06, all at reduced frequency *ω*/*ω*_*D*_ ≃ 0.2. Red and blue circles indicate phase singularities with winding numbers *q* = +1 and *q* = −1, respectively, and arrows show the normalized eigenvector components. Bottom panels: corresponding singularity density *N*_*TD*_/2*N* as a function of frequency for (d) the supercooled liquid, (e) the dodecagonal quasicrystal, and (f) the hexagonal crystal. The black line indicates a quadratic low-frequency reference trend, following the behavior reported for a two-dimensional glass in ref. 47.

Representative phase-field visualizations in Fig. 11 reveal distinct textures across the three systems. At *ω*/*ω*_D_ ≈ 0.2, the supercooled liquid shows an irregular phase pattern, while the crystal exhibits a more stripe-like structure. The DDQC lies between these limits, with a phase pattern that is less regular than that of the crystal but more organized than that of the liquid.

The corresponding singularity densities are shown in Fig. 11. At low reduced frequencies, all three systems show a similar increase in singularity density with frequency. At higher *ω*/*ω*_D_, the supercooled liquid exhibits a somewhat larger singularity density than the DDQC and the crystal, which remain quantitatively close over much of the plotted range. We interpret these results conservatively. The singularity count provides a geometric characterization of the eigenmode phase fields, but should not be considered as a structural order parameter or direct evidence of physically identifiable defects. In particular, the elevated high-frequency values in the supercooled liquid likely reflect a more disordered eigenmode phase pattern rather than stable defect-mediated transport.

For this reason, we consider this analysis complementary to the main vibrational observables, such as the vibrational density of states and participation ratio, rather than a primary means of distinguishing the three systems.

## Data Availability

The software and codes used to generate the data supporting the findings of this study are available at the Zenodo repository https://doi.org/10.5281/zenodo.16965771 with DOI 10.5281/zenodo.16965771.^67^ The repository contains scripts for running the molecular dynamic simulations as well as scripts for performing the subsequent data analysis.
